# The effect of combining an inhaled corticosteroid and a long-acting muscarinic antagonist on human airway epithelial cells in vitro

**DOI:** 10.1186/s12931-024-02710-8

**Published:** 2024-02-28

**Authors:** Maria Gabriella Matera, Barbara Rinaldi, Cecilia Calabrese, Carmela Belardo, Luigino Calzetta, Mario Cazzola, Clive Page

**Affiliations:** 1 Unit of Pharmacology, Department of Experimental Medicine, University of Campania “Luigi Vanvitelli”, Naples, Italy; 2 Department of Translational Medical Sciences, University of Campania “Luigi Vanvitelli”, Naples, Italy; 3 Respiratory Disease and Lung Function Unit, Department of Medicine and Surgery, University of Parma, Parma, Italy; 4 Unit of Respiratory Medicine, Department of Experimental Medicine, University of Rome “Tor Vergata”, Rome, Italy; 5 Pulmonary Pharmacology Unit, Institute of Pharmaceutical Science, King’s College, London, UK

**Keywords:** Airway epithelial cells, Asthma, Inhaled corticosteroid, Inflammation, Long-acting muscarinic antagonists

## Abstract

**Background:**

Airway epithelial cells (AECs) are a major component of local airway immune responses. Direct effects of type 2 cytokines on AECs are implicated in type 2 asthma, which is driven by epithelial-derived cytokines and leads to airway obstruction. However, evidence suggests that restoring epithelial health may attenuate asthmatic features.

**Methods:**

We investigated the effects of passive sensitisation on IL-5, NF-κB, HDAC-2, ACh, and ChAT in human bronchial epithelial cells (HBEpCs) and the effects of fluticasone furoate (FF) and umeclidinium (UME) alone and in combination on these responses.

**Results:**

IL-5 and NF-κB levels were increased, and that of HDAC-2 reduced in sensitised HEBpCs. Pretreatment with FF reversed the effects of passive sensitisation by concentration-dependent reduction of IL-5, resulting in decreased NF-κB levels and restored HDAC-2 activity. Addition of UME enhanced these effects. Sensitized HEBpCs also exhibited higher ACh and ChAT levels. Pretreatment with UME significantly reduced ACh levels, and addition of FF caused a further small reduction.

**Conclusion:**

This study confirmed that passive sensitisation of AECs results in an inflammatory response with increased levels of IL-5 and NF-κB, reduced levels of HDAC-2, and higher levels of ACh and ChAT compared to normal cells. Combining FF and UME was found to be more effective in reducing IL-5, NF-κB, and ACh and restoring HDAC-2 compared to the individual components. This finding supports adding a LAMA to established ICS/LABA treatment in asthma and suggests the possibility of using an ICS/LAMA combination when needed.

## Introduction

Airway epithelial cells (AECs), initially thought to act as a barrier against pathogens and inhaled allergens, play a key role in the development of asthma [1]. In AECs isolated from subjects with asthma and exposed to pathogens or allergens, there are inappropriate immune and inflammatory responses that disturb the airway epithelial layer. This is demonstrated by epithelial cell desquamation and loss of cell-cell contacts, culminating in reduced integrity and increased permeability [2]. AECs when exposed to harmful agents, release alarmins like thymic stromal lymphopoietin, interleukin (IL)-25, and IL-33 [3]. Alarmins trigger the proliferation of type 2 (Th2) inflammatory cells, which, in turn, secrete pro-inflammatory cytokines, including the pleiotropic cytokine IL-5. IL-5 plays a crucial role in the chemoattraction of eosinophils, as well as their proliferation, differentiation, survival, and activation, and also in the secretion of IL-4 and IL-13 [4]. Pro-inflammatory cytokines lead to a further increase in the release of alarmins [5]. As a result, targeting the epithelial airway barrier could be a promising new approach for treating asthma and other allergic conditions. Indeed, given that loss of epithelial barrier integrity is known to be a common feature of many chronic inflammatory diseases, targeting the respiratory epithelium has been suggested to be a plausible approach to treat such conditions [6].

Most patients with asthma respond well to a combination of inhaled corticosteroids (ICS) and bronchodilators. ICSs represent a first-line treatment in Th2 asthma patients, as they have anti-inflammatory effects by regulating cytokine production by immune cells [7]. However, it is unclear whether ICSs have direct beneficial effects on epithelial health or barrier function [8], although it has been noted that, the epithelium from subjects with asthma was less responsive to ICSs. Oxidative stress may contribute to this poor responsiveness of the epithelium to ICS by phosphoinositide-3‐kinase‐dependent post‐translational histone deacetylase (HDAC)-2 modifications and proteasomal HDAC-2 degradation [9].

Other research has also suggested that acetylcholine (ACh), acting via muscarinic receptors (mAChRs), plays a critical role in the pathophysiology of asthma [10]. It is well established that ACh activates M_3_ mAChRs on airway smooth muscle (ASM), leading to bronchoconstriction. However, it is also now appreciated that ACh can also contribute to inflammation and remodelling of the airways and regulate the growth of ASM [11]. Inflammatory stimuli induce ACh secretion from the AECs [12]. The airway epithelium expresses the ACh-synthesising enzyme choline acetyltransferase (ChAT), providing an immediate source of non-neuronal ACh, which is synthesised and secreted by non-innervated cells [12]. ACh release leads to autocrine or paracrine mAChR stimulation on AECs and surrounding cells, thereby increasing cholinergic tone in the asthmatic lung. In addition, it triggers pathophysiological events such as inflammation, remodelling and hypersecretion of mucus [13].

The ability of long-acting muscarinic antagonists (LAMAs) to effectively treat asthma is based on the critical function of ACh in the pathophysiology of asthma [13, 14]. The evidence that LAMAs have significant anti-inflammatory and anti-proliferative properties, as well as the ability to inhibit allergen-induced airway remodelling in animal or in vitro models, strongly suggests that LAMAs may have benefits beyond simply being bronchodilators.

According to the National Heart, Lung and Blood Institute/National Asthma Education and Prevention Program guidelines [15] and the Global Initiative for Asthma therapeutic strategy [16], the use of LAMAs as monotherapy in asthma should be avoided. However, experimental data indicates that combining ICS and LAMA results in a synergistic enhancement of bronchial relaxation in passively sensitised human medium and small bronchi [17]. This effect is linked to increased concentrations of cyclic adenosine monophosphate, an observation not seen in non-sensitised bronchi.

Passive sensitisation has been utilised as an in vitro model of bronchial asthma, which reflects important functional characteristics of non-specific airway inflammation and hyperresponsiveness present in individuals suffering from asthma [18, 19].

The current study evaluates the effects of passive sensitisation on IL-5, nuclear factor-κB (NF-κB), HDAC-2, ACh, and ChAT in human AECs. It also assesses the impact of fluticasone furoate (FF) and umeclidinium (UME) individually and in combination on these responses.

## Methods

### Cell culture

We utilised a stable Human Bronchial Epithelial Cell (HBEpC) line, isolated from human bronchi. HBEpC were cultured as monolayers in appropriate PromoCell Cell Growth Medium (Growth Medium -Ready-to-use- C-21,060) containing 100 IU/ml penicillin and amphotericin 1:1000 at 37 °C in a humidified incubator with 5% CO_2_ once they have reached > 70% confluency. Before starting experiments, confluent monolayers (2.5 10^5^ cells) were grown in 6-well plates (Corning Costar, Cambridge, MA).

### Measurement of HBEpC viability

A Cell Counting Kit-8 (Dojindo Molecular Technologies, Germany) was used to determine cell viability. The amount of the formazan dye, generated by the activities of dehydrogenases in cells, was considered directly proportional to the number of living cells. Cell viability was determined at 24 and 48 h according to the manufacturer’s instructions. The assay was performed using a microplate reader at 450 nm (Infinite 2000, TECAN, Switzerland).

### Sensitisation of HBEpCs with allergen

HBEpCs were passively sensitized for 18 h with a solution of a 2% sensitizing serum obtained from a pool of atopic asthma patient samples obtained during an exacerbation (total immunoglobulin [Ig]E 1,000 U/ml^− 1^ specific to common aeroallergens). Non-sensitizing serum was obtained from non-atopic donors (total IgE 45 U/ml^− 1^). The subjects provided signed consent for serum donation (ethical approval: R.S. 37/20, 2020; CEI, Independent Ethical Committee, Fondazione PTV Policlinico “Tor Vergata”, Italy). Sera were prepared from whole blood by centrifugation. Sera were frozen at -80 °C in 250 µl aliquots until needed and stored in small aliquots at -80 °C until used.

After 18 h, the cells were treated with FF (150 nM, 1.5 µM) or UME (128 nM, 1.28 µM) alone or in combination for 24 h. The drugs were diluted in distilled water and DMSO, the maximum amount of which did not affect the response of HBEpCs (2018). HBEpCs were then stored in small aliquots at -80 °C until they were used. FF and UME were tested at a concentration ratio that mimics that of the fixed dose combination currently approved for the treatment of adult asthmatic patients.

### Study design

The effect of FF and UME alone, and in combination, was evaluated on the activity of ChAT, ACh, NF-kB, HDAC-2 and IL-5 in non-sensitised and sensitised HBEpC.

### Quantification of IL-5, NF-kB, HDAC*-2*, ACh and ChAT

After the treatments, the supernatant from the HEBpC cultures was collected for the quantification of IL-5, NF-kB, I-kB-α, HDAC-2, ACh and ChAT release. Quantification was performed using colorimetric/fluorometric and ELISA assays characterised by high sensitivity detection limits and high specificity, in accordance with the manufacturers’ data sheets (Elabscience. Elabscience Laboratory Biological Research Reagents. www.elabscience.com). All study procedures were performed under blinded conditions, with both operator and data analysis blinded.

### Statistical analysis

Data were representative of 4 independent experiments and are shown as mean ± standard error. Analyses were performed by one-way analysis of variance (ANOVA) to compare differences among multiple groups, followed by Student’s *t* test to distinguish differences between two groups. A *p* value ≤ 0.05 was considered as significant. All data analyses were performed using computer software GraphPad Prism 8 (GraphPad Software, La Jolla, CA, USA).

## Results

### Effects of FF and UME, alone or in combination, on IL-5, NF-kB and HDAC-2 in non-sensitized and sensitized HEBpCs

**Fig. 1:**
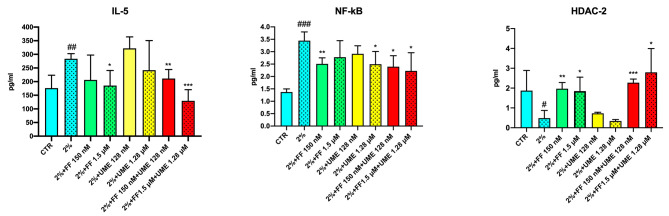
Effect of fluticasone furoate (FF) and umeclidinium (UME) at different concentrations, alone or in combination, on interleukin-5 (IL-5), nuclear factor-κB (NF-kB) and histone deacetylase-2 (HDAC-2) levels (pg/ml) in sensitized human bronchial epithelial cells (2%). Non-sensitized cells (CRT) are control. Values represents the mean ± SEM of four samples per group. * *P* < 0.05, ** *P* < 0.01, *** *P* < 0.001 vs. sensitized cells; # *P* < 0.05, ### *P* < 0.001 vs. control cells

Figure 1; Table 1 show the effects of FF and UME on IL-5, NF-kB and HDAC-2 levels.

**Table 1:** Changes in interleukin-5 (IL-5), nuclear factor-κB (NF-kB) and histone deacetylase-2 (HDAC-2) levels (pg/ml) from pretreatment values induced by fluticasone furoate (FF) and umeclidinium (UME) at different concentrations, alone or in combination, in non-sensitised and sensitised human bronchial epithelial cells (HBEpCs).

	Non-sensitized HEBpCs							Sensitized HEBpCs						
	Pretreatment	FF 150 nM	FF 1.5 µM	UME 128 nM	UME 1.28 µM	FF 150 + UME 128 nM	FF1.5 + UME 1.28 µM	Pretreatment	FF 150 nM	FF 1.5 µM	UME 128 nM	UME 1.28 µM	FF 150 + UME 128 nM	FF1.5 + UME 1.28 µM
IL-5	176.1 ± 23.7	-9.77 ± 8.5	-9.52 ± 3.1	-9,52 ± 9.6	-13.63 ± 4.7	-15.33 ± 7.5	-10.4 ± 3.3	283.9 ± 9.4	-77.6 ± 46.5	-98.3 ± 29.2 *	+ 37.9 ± 23.2	-41.8 ± 55.0	-72.4 ± 18.9 **	-154.3 ± 22.5 ***
NF-kB	1.37 ± 0.06	-0.02 ± 0.04	-0.05 ± 0.03	-0.05 ± 0.03	-0.03 ± 0.03	-0.06 ± 0.03	-0.08 ± 0.02	3.44 ± 0.18	-0.94 ± 0.21 **	-0.67 ± 0.38	-0.53 ± 0.24	-0.95 ± 0.31 *	-1.05 ± 0.28 *	-1.22 ± 0.40 *
HDAC-2	1.87 ± 0.51	+ 0.17 ± 0.04	+ 0.18 ± 0.06	+ 0.02 ± 0.09	+ 0.10 ± 0.03	+ 0.11 ± 0.09	+ 0.19 ± 0.09	0.48 ± 0.22	+ 1.49 ± 0.25 **	+ 1.36 ± 0.40 *	+ 0.24 ± 0.20	-0.14 ± 0.20	+ 1.79 ± 0.21 ***	+ 2.31 ± 0.63 *

* *P* < 0.05, ** *P* < 0.01, *** *P* < 0.001 vs. pretreatment values

IL-5 levels were found to be significantly higher (*p* < 0.01) in sensitised HEBpCs (283.9 ± 9.4 pg/ml) than in non-sensitised HEBpCs (176.1 ± 23.7 pg/ml). Treatment of non-sensitised HEBpCs with FF and UME alone and in combination did not alter IL-5 levels. Conversely, in sensitised HEBpCs, pretreatment with FF reduced IL-5 levels in a concentration-dependent manner, whereas UME reduced IL-5 levels only at high concentrations. FF combined with UME at low concentrations reduced IL-5 levels (-72.41 ± 18.94 pg/ml, *p* < 0.01). At high concentrations, the combination caused a further reduction in IL-5 that was numerically greater than that observed with the individual treatments (-56.0 ± 34.4 pg/ml vs. FF 1.5 µM, ns; -112.5 ± 57.9 pg/ml vs. UME 1.28 µM, ns), but also than that induced by the combination at low concentrations (-81.9 ± 26.2 pg/ml, *p* < 0.05).

A significant (*p* < 0.001) increase in NF-kB expression levels (3.44 ± 0.18 pg/ml) was found in sensitised HEBpCs compared to NF-kB levels in non-sensitised HEBpCs (1.37 ± 0.06 pg/ml). Treatment of non-sensitised HEBpCs with FF and UME alone and in combination did not change NF-kB levels compared to sensitised HEBpCs. Pretreatment of sensitised HEBpCs with FF reduced NF-kB levels, but the reduction was consistent and statistically significant only at the low concentration of FF. UME also reduced NF-kB levels, but to a lesser extent than FF. The combination of FF + UME also reduced NF-kB levels in sensitised HEBpCs. The combination caused a further numerical reduction in NF-kB levels compared to FF (FF 150 nM + UME 128 nM: -0.11 ± 0.25 pg/ml, ns vs. FF 150 nM; FF 1.5 µM + UME 1.28 µM: -0. 56 ± 0.50 pg/ml, ns vs. FF 1.5 µM) and UME (FF 150 nM + UME 128 nM: -0.52 ± 0.28 pg/ml, ns vs. UME 128 nM; FF 1.5 µM + UME 1.28 µM: -0.27 ± 0.45 pg/ml, ns vs. UME 1.28 µM).

Sensitisation of HEBpCs resulted in significantly (*p* < 0.05) lower levels of HDAC-2 (0.48 ± 0.22 pg/ml) than in non-sensitised HEBpCs (1.87 ± 0.51 pg/ml). Treatment of non-sensitised HEBpCs with FF and UME alone and in combination did not alter HDAC-2 levels. In contrast, pretreatment of sensitised HEBpCs with FF increased HDAC-2 levels; UME was ineffective compared to FF in increasing HDAC-2 levels under the same experimental conditions. However, the combination of FF and UME at low and high concentrations induced significant increases in pretreatment HDAC-2 levels. Both combinations induced only numerical increases compared to the corresponding FF (FF 150 nM + UME 128 nM: -0.30 ± 0.18 pg/ml, ns vs. FF 150 nM; FF 1.5 µM + UME 1.28 µM: -0.95 ± 0.70 pg/ml, ns vs. FF 1.5 µM).

### Effect of FF and UME alone, or in combination, on ACh and ChAT in non-sensitized and sensitized HEBpCs

**Fig. 2:**
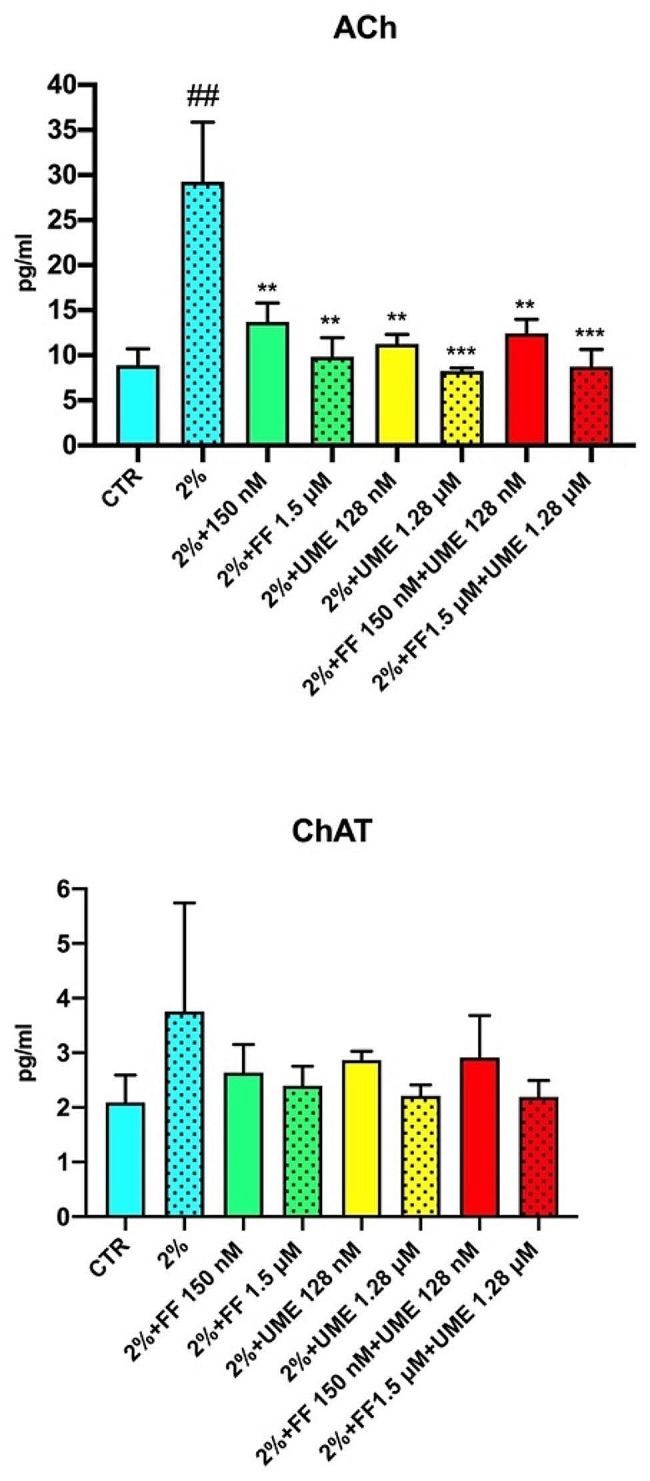
Effect of fluticasone furoate (FF) and umeclidinium (UME) at different concentrations, alone or in combination, on acetylcholine (ACh) and choline acetyltransferase (ChAT) levels (pg/ml) in sensitized human bronchial epithelial cells (2%). Non-sensitized cells (CRT) are control. Values represents the mean ± SEM of four samples per group. * *P* < 0.05, ** *P* < 0.01, *** *P* < 0.001 vs. sensitized cells; # *P* < 0.05, ### *P* < 0.001 vs. control cells

Figure 2; Table 2 show the effects of FF and UME on ACh and ChAT levels in sensitised HEBpCs.

**Table 2:** Changes in acetylcholine (ACh) and choline acetyltransferase (ChAT) levels (pg/ml) from pretreatment values induced by fluticasone furoate (FF) and umeclidinium (UME) at different concentrations, alone or in combination, in non-sensitised and sensitised human bronchial epithelial cells (HBEpCs)

	Non-sensitized HEBpCs							Sensitized HEBpCs						
	Pretreatment	FF 150 nM	FF 1.5 µM	UME 128 nM	UME 1.28 µM	FF 150 + UME 128 nM	FF1.5 + UME 1.28 µM	Pretreatment	FF 150 nM	FF 1.5 µM	UME 128 nM	UME 1.28 µM	FF 150 + UME 128 nM	FF1.5 + UME 1.28 µM
ACh	8.90 ± 0.91	+ 0.12 ± 0.70	+ 0.59 ± 0.66	+ 0.96 ± 0.84	-0.56 0.41±	+ 0.75 ± 0.55	+ 0.57 ± 0.93	29.25 ± 3.30	-15.52 ± 3.46 **	-19.41 ± 3.47 **	-17.99 ± 3.34 **	-20.98 ± 3.31 ***	-16.82 ± 3.39 **	-20.50 ± 3.44 ***
ChAT	2.09 ± 0.25	+ 0.05 ± 0.21	-0.03 ± 0.13	+ 1.40 ± 0.33	-0.02 ± 0.18	-0.01 ± 0.08	-0.13 ± 0.10	3.76 ± 1.00	-1.12 ± 1.02	-1.36 ± 1.01	-0.89 ± 1.00	-1.55 ± 1.00	-0.84 ± 1.06	-1.57 ± 1.00

* *P* < 0.05, ** *P* < 0.01, *** *P* < 0.001 vs. pretreatment values

The results show that sensitised HEBpCs exhibited higher levels of ACh and ChAT (ACh: 29.25 ± 3.30 pg/ml; ChAT: 3.76 ± 1.00 pg/ml) compared to non-sensitised HEBpCs (ACh: 8.90 ± 0.91 pg/ml, *p* < 0.01; ChAT: 2.09 ± 0.25 pg/ml, ns). ACh and ChAT levels in non-sensitised HEBpC were not affected by treatment with FF and UME alone or in combination.

Pre-treatment of sensitised HEBpCs with FF or UME, alone or in combination, resulted in significantly reduced ACh levels, but the combination did not provide any advantages over the mono-components (FF 150 nM + UME 128 nM: -1.29 ± 1. 30 pg/ml, ns vs. FF 150 nM; FF 1.5 µM + UME 1.28 µM: -1.09 ± 1.43 pg/ml, ns vs. FF 1.5 µM) and UME (FF 150 nM + UME 128 nM: 1. 17 ± 0.95 pg/ml, ns vs. UME 128 nM; FF 1.5 µM + UME 1.28 µM: 0.48 ± 0.97 pg/ml, ns vs. UME 1.28 µM).

Contrary to what was observed with ACh levels, the decrease in ChAT levels induced by FF and UME, even when used in combination, was always statistically insignificant.

## Discussion

In this study we have confirmed that passive sensitisation of AECs leads to an inflammatory response with increased levels of IL-5 and NF-κB and decreased levels of HDAC-2. Activation of NF-κB by IL-5 amplifies the inflammatory response and has been suggested to then contribute to reducing the anti-inflammatory effect of corticosteroids, as recruitment of HDAC-2 is known to effectively inhibit all activated inflammatory genes [20].

As previously discussed, corticosteroids are recognised as drugs that can target the airway epithelium as part of their anti-inflammatory actions. Numerous studies have shown that regular treatment with ICS restores epithelial integrity while also significantly reducing eosinophils [21–24]. Our observations indicate that pretreatment with FF effectively reversed the effects of passive AEC sensitisation through a concentration-dependent reduction in IL-5. This reduction in IL-5 subsequently reduced NF-κB and restored HDAC-2 activity, confirming the anti-inflammatory effect of ICS on airway epithelial cells. Furthermore, our study shows that FF reduced both ACh and ChAT levels in sensitised HBEpCs, although the decrease in ChAT was only numerical and not statistically significant. These findings are consistent with those of Reinheimer et al. [25] who demonstrated in a rat model of asthma that dexamethasone reduced epithelial ACh by approximately 80% and inhibited epithelial ChAT activity.

When analysing the effect of UME in this in vitro model, no significant effect on IL-5 was observed at low concentrations. However, higher concentrations led to a reduction in IL-5 and were able to modify levels of NF-κB and HDAC-2. These results are consistent with the pro-inflammatory action of non-neurogenic ACh [10, 13, 14].

Several non-clinical experimental studies have documented the anti-inflammatory effects of LAMAs in the airways. LAMAs can control airway contractility and hyperresponsiveness through antagonism of mAChRs on ASM, as well as anti-inflammatory mechanisms that block mAChRs on inflammatory cells, sub-mucosal glands, and epithelial cells [26]. Much of the current information suggesting a possible anti-inflammatory action of LAMAs has been obtained using tiotropium bromide. Tiotropium has been reported to reduce cytokine and chemokine synthesis and release, as well as the number of inflammatory cells, both in vivo and in vitro models. Furthermore, tiotropium significantly reduced airway inflammation and remodeling in an allergic mouse model and improved lung function [27]. Tiotropium has also been reported to reduce eosinophilic inflammation in chronically challenged allergic guinea pigs to a similar extent to budesonide [28]. Tiotropium has also been demonstrated to inhibit the enhancement of NF-κB activity in an in vitro murine model of COPD involving BEAS-2B human bronchial epithelial cells that have been immortalized [29]. There is also evidence showing that tiotropium minimised the excessive release of IL-5 and IL-13 in human peripheral blood mononuclear cells derived from asthma patients [30, 31]. Also, at high dosages, tiotropium decreased the total number of inflammatory cells, including macrophages and eosinophils, and the levels of transforming growth factor-β1, IL-4, IL-5, and IL-13 in bronchoalveolar lavage fluid in a mouse model of asthma [30].

Our current research has also demonstrated that pretreating sensitised AECs with UME led to a reduction in ACh and ChAT, which can presumably be attributed to the decreased levels of IL-5 and NF-κB seen after treatment of the cells with this drug. This effect further supports the suggestions that UME has an anti-inflammatory action.

In contrast to the considerable amount of research on the links between ICS and the adrenergic system, there is less evidence investigating the interactions between ICS and the cholinergic system. Nevertheless, the airway epithelium is a major source of non-neuronal ACh due to its ChAT expression, which can be enhanced by inflammatory stimuli, resulting in increased ACh synthesis and hence cholinergic effects in the airways [10, 12]. In our in vitro model, there was a statistically significant increase in ACh levels and a 79.9% numerical, although not statistically significant, increase in ChAT levels in sensitised AECs, supporting a role for these cells in diseases such as asthma, which are known to be associated with epithelial dysfunction [6]. Incubation with immunoglobulin (Ig)E has previously been shown to facilitate cholinergic function in human airways [32]. Furthermore, in a murine asthma model, IgE was found to increase ACh levels compared to controls [33]; subsequently, ACh stimulated epithelial inflammatory responses by activating mAChRs on epithelial cells. This finding suggests the need to consider targeting ACh in the treatment of asthma by including a LAMA in the management of such patients.

The combination of FF and UME was found to be more effective in reducing IL-5, NF-κB and HDAC-2 levels compared to the individual components. These findings support previous reports demonstrating the potential anti-inflammatory effects of the combination of ICS and LAMA in in vitro models [17]. Furthermore, administration of tiotropium and ciclesonide in an animal model of asthma showed inhibition of inflammation and airway remodelling [34], indicating the potential clinical benefit of a combination of an ICS and LAMA.

Finally, the combination of FF and UME caused a greater reduction in ACh and ChAT levels in sensitised AECs compared to normal cells, suggesting a potential additional pharmacological interaction between corticosteroids and mAChRs antagonists, which supports the rationale for the use of ICS/LAMA as a treatment approach for subjects with asthma and strengthens the pharmacological rationale for adding a LAMA to the established use of a combination of long-acting b-agonist/ICS in the context of “triple therapy”.

It should also be noted that emerging evidence suggests that LAMAs in combination with an ICS may have a role both in the treatable trait approach, recommended as a new paradigm for asthma management [35], and in the treatment of asthmatic smokers who have more severe obstructive impairment compared with non-smokers and light smokers on equivalent doses of ICS [36]. However, further studies are needed to better characterise experimentally the role of LAMAs in the ultimate control of airway inflammation and the true extent of their interaction with corticosteroids.

## Conclusion

This study confirmed that passive sensitisation of AECs results in an inflammatory response with increased levels of IL-5 and NF-κB, reduced levels of HDAC-2 and higher levels of ACh and ChAT compared to normal cells. The combination of FF and UME was found to be more effective in reducing IL-5, NF-κB, and ACh and restoring HDAC-2 than the individual components. This finding supports the addition of a LAMA to established ICS/LABA treatment in asthma and suggests the possibility of using an ICS/LAMA combination when needed.

## Data Availability

All data will be made available by the authors upon reasonable request.

## Abbreviations

Ach: acetylcholine
AECs: airway epithelial cells
ASM: airway smooth muscle
ChAT: choline acetyltransferase
FF: fluticasone furoate
HBEpC: Human Bronchial Epithelial Cell
HDAC: histone deacetylase
ICS: inhaled corticosteroid
Ig: immunoglobulin
IL: interleukin
LAMA: long-acting muscarinic antagonist
mAChR: muscarinic receptors
NF-κB: nuclear factor-κB
Th2: type 2
UME: umeclidinium
